# A Comprehensive Analysis of Adverse Events Associated with HER2 Inhibitors Approved for Breast Cancer Using the FDA Adverse Event Report System (FAERS)

**DOI:** 10.3390/ph18101510

**Published:** 2025-10-08

**Authors:** Airi Yajima, Yoshihiro Uesawa

**Affiliations:** Department of Medical Molecular Informatics, Meiji Pharmaceutical University, 2-522-1 Noshio, Kiyose, Tokyo 204-8588, Japan

**Keywords:** HER2 inhibitors, adverse event profile, molecular targeted therapy, breast cancer, FAERS database, principal component analysis, cluster analysis, monoclonal antibody, antibody–drug conjugate, tyrosine kinase inhibitor

## Abstract

**Background/Objectives:** Human epidermal growth factor receptor 2 (HER2) inhibitors have markedly improved outcomes in patients with HER2-positive breast cancer. Clinical treatment often involves the sequential or combined use of multiple HER2 inhibitors, making it essential to clarify their distinct adverse event (AE) profiles. However, AE trends remain insufficiently understood. This study aimed to comprehensively analyze characteristic AEs associated with HER2 inhibitors. **Methods:** Using the U.S. Food and Drug Administration Adverse Event Reporting System (FAERS, January 2004–September 2024), we conducted disproportionality analyses of AEs associated with HER2 inhibitors approved for breast cancer. Based on the natural logarithm of the reporting odds ratio (lnROR), hierarchical cluster analysis and principal component analysis (PCA) were performed. **Results:** Disproportionality analysis treating HER2 inhibitors as a single group identified several signals, with hair disorder (ROR 39.93 [95% CI: 37.68–42.32]) as a representative example. Hierarchical clustering showed that monoclonal antibodies (mAbs) and tyrosine kinase inhibitors (TKIs) diverged early in the dendrogram, and clusters broadly corresponded to pharmacological classes. The cluster of hair-related AEs closely corresponded to mAbs. PCA indicated that the first component reflected AE occurrence risk (R^2^ = 0.655, *p* < 0.0001), the second component distinguished mAbs from TKIs (tucatinib: r = 0.667; trastuzumab: r = −0.567), and the third component separated molecular targeted agents from antibody–drug conjugates (neratinib: r = 0.521; T-DXd: r = −0.440). **Conclusions:** FAERS-based analyses enabled visualization of the distinct AE profiles of HER2 inhibitors. These findings may support safe drug selection, risk stratification, and improved AE management strategies.

## 1. Introduction

Human epidermal growth factor receptor 2 (HER2) is a membrane-bound receptor-type tyrosine kinase encoded by *ERBB2*. It belongs to the HER family, which comprises HER1, HER2, HER3, and HER4 [1]. In healthy cells, HER2 receptors are involved in signal transduction processes like cell proliferation and differentiation. However, preclinical studies suggest that HER2 protein overexpression is associated with enhanced tumor cell proliferation and increased metastatic potential, indicating possible involvement in malignancy. HER2 overexpression is observed in various cancers, particularly breast [2] and gastric cancers [3], with an expression rate of 10–35% in breast cancer patients [2]. Shortened disease-free survival, increased recurrence rates, and significantly reduced overall survival have been reported in this population, indicating poor prognosis [4].

Following the development of trastuzumab [5,6,7], recently, molecular targeted agents directed against HER2 and other HER family receptors have been developed and introduced into clinical practice. Agents inhibiting HER2 activity provide markedly improved outcomes for patients with HER2-positive breast cancer and play an essential role in treatment strategies. Whereas these agents all inhibit HER2 activity, certain drugs also target other members of the EGFR family, resulting in diverse mechanisms of action and safety profiles. In treating HER2-positive breast cancer, targeted therapy is recommended using HER2 inhibitors combined with chemotherapy regardless of disease stage [8], with well-established efficacy [5,6,7,9,10,11,12,13,14]. In treatment models where several HER2-targeted agents are administered sequentially, a comprehensive understanding of the associated adverse events (AEs) is vital for optimal side effect management. Therefore, a holistic understanding of the safety profiles of HER2 inhibitors is needed.

Representative AEs reported for HER2 inhibitors include cardiotoxicity and infusion reactions from monoclonal antibodies (mAbs) like trastuzumab [6,15]; thrombocytopenia from antibody–drug conjugates (ADCs) like trastuzumab emtansine (T-DM1) [16]; interstitial lung disease (ILD) associated with trastuzumab deruxtecan (T-DXd) [11]; and diarrhea from tyrosine kinase inhibitors (TKIs) like neratinib [14]. However, comprehensive investigations remain limited on the overall AEs associated with these agents.

To holistically evaluate the AEs associated with HER2 inhibitors, we examined large-scale spontaneous reporting systems (SRSs) data. SRSs are valuable data sources for post-marketing safety surveillance, enabling the detection of rare side effects that may not emerge during clinical trials. The Food and Drug Administration (FDA) Adverse Event Reporting System (FAERS), maintained by the United States FDA, contains a multinational dataset. Its large volume of reports allows for highly accurate statistical analyses, contributing to a more precise understanding of AE trends.

In addition to conventional disproportionality analyses, this study incorporated two complementary multivariate data mining approaches—hierarchical cluster analysis [17] and principal component analysis (PCA) [18]—to explore the complex relationships between HER2-targeted agents and their associated AEs. Cluster analysis groups drugs or events according to similarities in their safety profiles, revealing latent patterns that may not be apparent from univariate measures alone. PCA reduces the dimensionality of large, correlated datasets by transforming them into orthogonal components, facilitating the visualization and interpretation of overarching trends while retaining most of the original information. Applying these methods to large-scale spontaneous reporting system data enables a more comprehensive characterization of AE patterns across HER2 inhibitors, complementing traditional signal detection and supporting a nuanced understanding of their safety profiles.

The objective of this study was to comprehensively evaluate the major AEs related to HER2 inhibitors approved for breast cancer in the FAERS database, employing cluster analysis and PCA. Our findings are expected to enhance our understanding of AE occurrence patterns and safety profiles, informing optimal drug selection and improving AE management.

## 2. Results

### 2.1. Baseline Characteristics of the Analytic Dataset

The analytic dataset comprised 96,222 patients. Data on sex were available for 82,416 (85.6%) patients; among these, 76,736 (93.1%) were female and 5108 (6.2%) were male, with 572 (0.7%) unknown. Age was recorded for 57,608 individuals, yielding a median of 57 years (IQR 48–66); the largest age group was 50–59 years (17,031; 29.6%). Data on weight were available for 27,265 patients, with a median weight of 65 kg (IQR 56–78), and 7140 (26.2%) patients weighed 60–69 kg. Most reports originated from the United States (37,984; 39.7%), followed by China, Japan, the United Kingdom, and Canada (Table 1).

### 2.2. Construction of the Analytical Data Table

**Figure 1:**
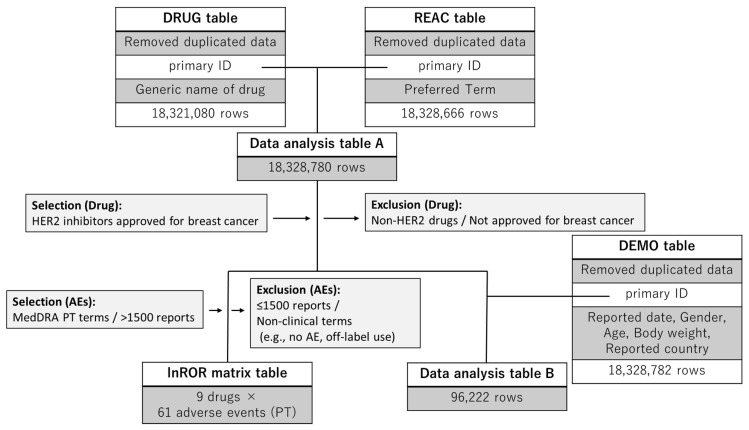
Flowchart for data analysis table construction. The reporting odds ratio (ROR)-calculator generated the data analysis table and the lnROR matrix. The data analysis table A is used to examine adverse event reports, calculate *p*-values, and report RORs and 95% confidence intervals for drugs and adverse reactions. The lnROR matrix was used for hierarchical clustering and PCA. Data analysis table B was used to compile baseline demographic and clinical characteristics of patients. The DRUG, REAC, and DEMO tables were merged using a full outer join on the primary ID. Subsequently, records with missing values in either the DRUG or REAC table were excluded to ensure the consistency of the analytical dataset.

We created a combined data table by merging the FAERS drug information (DRUG) table, which comprises 18,321,080 rows, the AE information (REAC) table, which comprises 18,328,666 rows, and the demographic data (DEMO) table, which comprises 18,328,782 rows. Duplicate entries were removed during the table construction process. A flowchart illustrating the table construction process is presented in Figure 1.

### 2.3. Number of Reports

Table 2 presents the number of AEs reported for the nine HER2 inhibitors included in the FAERS database.

### 2.4. Frequently Reported AEs

Sixty-three AEs with >1500 reported cases linked to the examined HER2 inhibitors were identified. After excluding two preferred terms (“no adverse event” and “off-label use”), 61 AE categories were extracted and are displayed in Table S1. This table includes the AE term, the number of reports, ROR, 95% confidence interval, *p*-value, and the four-cell layout of the 2 × 2 contingency table used for ROR calculation. The cells are defined as follows: (a) AE with the target drug, (b) no AE with the target drug, (c) AE with a nontarget drug, and (d) no AE with a nontarget drug. This arrangement enables quantitative estimation of the association strength between each drug and AE via the ROR. Among these, diarrhea was most frequently reported, followed by nausea and fatigue. AEs with notably high ROR values (ROR [95% CI]) included: hair disorders, 39.93 [37.68–42.32]; metastases to the central nervous system, 38.96 [37.03–40.99]; decreased ejection fraction, 35.47 [33.87–37.15]; and cardiotoxicity, 34.46 [32.33–36.72]. These AEs were estimated to exhibit relatively strong associations with the HER2-targeting agents.

### 2.5. Hierarchical Cluster Analysis

**Figure 2:**
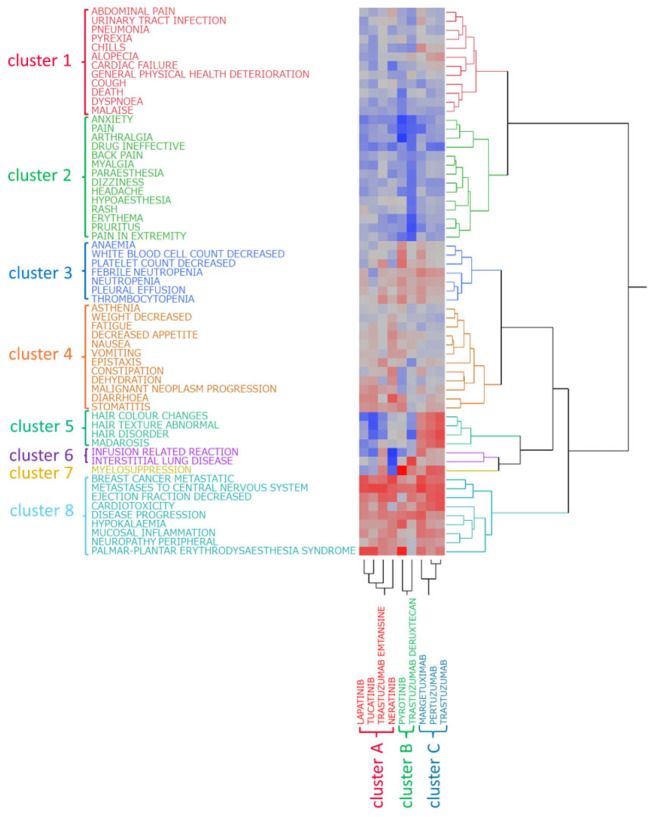
Hierarchical clustering analysis. This figure illustrates the relationship between nine drugs (three mAbs, two ADCs, and four TKIs) and the top 61 adverse events. In the colormap, red indicates a positive correlation, whereas blue represents a negative correlation.

Hierarchical cluster analysis (Ward’s method) was conducted on the nine HER2 inhibitors based on the lnROR values and AEs identified in Section 2.4. This analysis classified the drugs into three clusters (Figure 2): cluster A (lapatinib, neratinib, tucatinib, and T-DM1); cluster B (pyrotinib and T-DXd); and cluster C (trastuzumab, pertuzumab, and margetuximab). Similarly, hierarchical cluster analysis was performed on the 61 AEs, identifying eight distinct clusters (Figure 2): cluster 1 (including abdominal pain, urinary tract infection, and pneumonia); cluster 2 (including anxiety, pain, arthralgia, and back pain); cluster 3 (anemia, decreased white blood cell count, and decreased platelet count); cluster 4 (including asthenia, decreased weight, and fatigue); cluster 5 (hair color changes, abnormal hair texture, hair disorders, and madarosis); cluster 6 (infusion-related reactions and ILD); cluster 7 (myelosuppression); and cluster 8 (including metastatic breast cancer, metastases to the central nervous system, and decreased ejection fraction).

Notably, aside from the ADCs, the TKIs and mAbs separated early in the clustering process, indicating that their AE reporting profiles were clearly distinguished.

Furthermore, correspondence was observed between drug clusters and AE clusters. Cluster A (mainly TKIs and T-DM1) showed strong associations with cluster 4 (diarrhea, stomatitis, and dehydration). Cluster B (pyrotinib and T-DXd) was associated with cluster 3 (anemia, decreased white blood cell count, and decreased platelet count) and cluster 7 (myelosuppression). Cluster C (mAbs) was closely related to cluster 5 (hair-related AEs) and cluster 6 (infusion-related reactions and ILD).

The resulting clusters were divided based on the pharmacological classification of the drugs: cluster A primarily consisted of intracellularly acting TKIs and T-DM1, cluster B included pyrotinib and T-DXd, and cluster C consisted of mAbs targeting HER2. This consistency in classification suggests that the tendency for AEs (safety profile) of HER2 inhibitors may be closely related to the mechanism of action of the drug.

### 2.6. PCA

**Figure 3:**
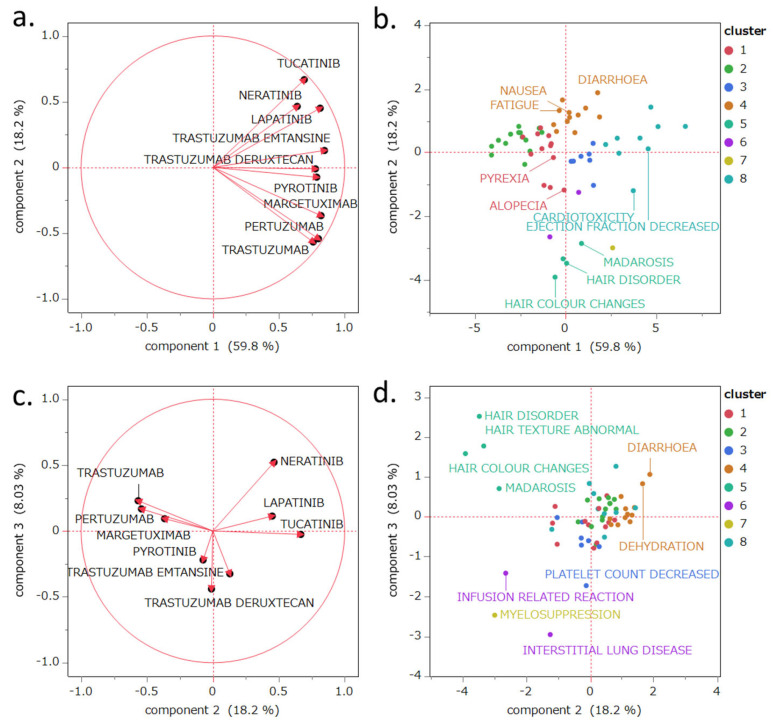
PCA of the evaluated drugs and major adverse events (AEs). The loading plots (**a**,**c**) show the relationships between the drugs and principal components, with each vector representing a drug. The score plots (**b**,**d**) illustrate the relationships between AEs and the principal components, with each dot representing an AE. The score plots (**b**,**d**) are color-coded according to clusters classified by hierarchical cluster analysis based on the results of Section 2.5. Panel (b): Labels were assigned to the top five preferred terms (PTs) with the highest number of reports and reporting odds ratios (RORs), excluding those related to disease progression. The most frequently reported events were diarrhea, nausea, fatigue, alopecia, and dyspnea. Events with the highest RORs included hair disorder, decreased ejection fraction, cardiotoxicity, madarosis, and hair color change. Panel d: PCA score plot. Labels were added to samples that were prominently distributed along both the positive and negative directions of the PC2 and PC3 axes.

**Figure 4:**
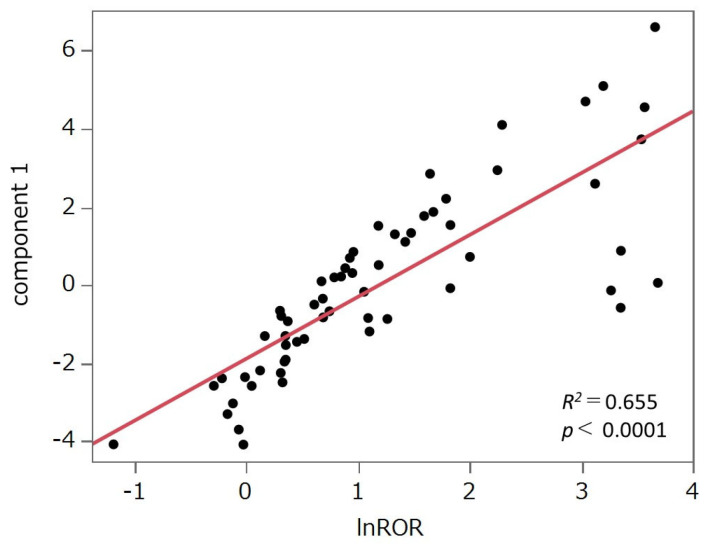
Scatter plot of principal component 1 and the natural log-transformed reporting odds ratio (lnROR). We evaluated the correlation between principal component 1 and lnROR, which was derived by applying a natural logarithmic transformation to the ROR calculated from all analyzed drugs treated as a single group of HER2 inhibitors. The drugs were plotted, and a least squares regression line was fitted to the data.

The PCA results are shown in Figure 3. The contribution rates of the first, second, and third principal components were 59.8%, 18.2%, and 8.03%, respectively. Principal component 1 demonstrated a positive correlation with the lnROR values calculated by collectively evaluating the target drugs as a single group of HER2 inhibitors (R^2^ = 0.655, *p* < 0.0001; see Figure 4). Principal component 2 was positively correlated with lapatinib (r = 0.450), neratinib (r = 0.464), and tucatinib (r = 0.667), whereas it exhibited negative correlations with pertuzumab (r = −0.542), trastuzumab (r = −0.567), and margetuximab (r = −0.367). Principal component 3 was positively correlated with neratinib (r = 0.521) and negatively correlated with T-DXd (r = −0.440) and T-DM1 (r = −0.325).

## 3. Discussion

### 3.1. Baseline Characteristics and Number of AE Reports Related to HER2 Inhibitors

The baseline characteristics of the analytic dataset, as summarized in Table 1, provide important clinical context. The predominance of female patients (93.1%) was consistent with the epidemiology of HER2-positive breast cancer [4,19]. In general, sex differences are known to influence drug metabolism and inflammatory pathways through variations in sex hormones and immune function, potentially leading to differences in efficacy and occurrence of adverse drug reactions [20]. Therefore, caution should be exercised while generalizing the interpretation of safety outcomes [21]. The median age was 57 years, with the largest subgroup being 50–59 years, aligning with the peri-menopausal period, during which pharmacokinetics and tolerability may vary. Notably, approximately 16% of patients were aged 70 years or older, a subgroup at elevated risk of trastuzumab-related cardiotoxicity and other adverse outcomes, underscoring the importance of careful monitoring in elderly patients [22]. The median body weight was 65 kg, which may reflect the contribution of reports from Asian countries and suggests a smaller body size compared with Western cohorts. Since body size influences drug exposure, especially for weight-based dosing regimens and fixed-dose ADCs, under- or over-exposure could potentially contribute to variations in toxicity risk. Geographically, nearly 40% of reports originated from the United States, but substantial contributions from China and Japan indicate regional variability in drug utilization and reporting behaviors. These differences should be considered when interpreting safety signals from FAERS [23].

Among the HER2 inhibitors analyzed, mAbs had the highest number of reported AEs, followed by TKIs and ADCs, as shown in Table 2. Notably, trastuzumab had the highest number of reports, which may be attributed to its long-standing clinical use.

Of the top 61 AEs selected for cluster analysis and PCA, diarrhea was the most frequent, followed by nausea and fatigue (Table S1). Previous studies also identified nausea and fatigue as common AEs associated with HER2 inhibitors [23]. Diarrhea was predominantly reported with HER2 TKIs [23,24].

In a previous study [23], AEs such as a decreased ejection fraction and cardiac failure were among the top 10 reported for trastuzumab. These were ranked 19th and 60th, respectively, in the current analysis. Similarly, at rank 38, thrombocytopenia, frequently reported with T-DM1, appeared as platelet count decreased, whereas ILD, a serious clinical concern with T-DXd, was ranked 40th. These findings suggest that the AEs identified by report frequency were appropriately selected for inclusion in the analysis.

### 3.2. Classification of HER2 Inhibitors Based on Hierarchical Clustering Analysis

Cluster analysis is a data mining technique that groups data based on similarities. Hierarchical clustering organizes data into nested clusters and generates dendrograms for visual representation.

In this study, Ward’s method was used to perform hierarchical clustering analysis on the nine HER2 inhibitors based on lnROR values calculated for 61 AE terms extracted in Section 4 (Materials and Methods). This analysis classified the drugs into three distinct clusters (Figure 2). Notably, aside from the ADCs, the TKIs and mAbs were separated early in the clustering process.

The early divergence of mAbs and TKIs observed in the dendrogram suggests that their AE profiles are fundamentally different. Pharmacologically, mAbs target extracellular HER2 receptors [25], exerting effects primarily through immune-mediated mechanisms and receptor blockade, whereas TKIs inhibit the intracellular kinase domain [26], directly blocking downstream signaling pathways. These mechanistic differences are likely to contribute to the distinct AE patterns detected in our clustering analysis, supporting the notion that pharmacological mode of action is closely linked with safety profiles.

Cluster A included lapatinib, neratinib, tucatinib, and T-DM1, with most agents being TKIs. This cluster exhibited strong associations with AEs, including fatigue, vomiting, and dehydration. Diarrhea—the most frequently reported AE—was associated with multiple drugs in this cluster. Diarrhea is a well-known common AE of TKIs [24] that can lead to substantial fluid and electrolyte loss, which may induce dehydration. Notably, all agents in cluster A, except T-DM1, showed poor associations with infusion-related reactions and myelosuppression, possibly due to their oral administration and the selective targeting of HER2, which has low expression in hematopoietic cells [25].

Cluster B included pyrotinib and T-DXd. Compared to the other clusters, these agents showed relatively poor associations with central nervous system-related AEs like anxiety and pain. However, both drugs showed similar associations with metastatic breast cancer and central nervous system metastases, suggesting that despite differing mechanisms, they may share common influences on disease metastasis. The limited associations with anxiety and pain imply a potentially lower impact on patients’ quality of life (QoL), which may be clinically advantageous.

Pyrotinib, an irreversible TKI targeting EGFR, HER2, and HER4 [27], was approved in 2018 in China and is widely used as a second-line therapy for HER2-positive metastatic breast cancer, especially in developing countries lacking access to innovative ADCs such as T-DM1 and T-DXd [28]. Systematic reviews and meta-analyses have confirmed its efficacy, including benefits for patients with brain metastases [29]. Similarly, T-DXd shows significantly improved progression-free survival in HER2-positive breast cancer patients with brain metastases [30].

Cluster C consisted exclusively of mAbs: trastuzumab, pertuzumab, and margetuximab. This cluster was strongly associated with hair-related AEs, including hair color changes, hair disorders, abnormal hair texture, and madarosis. Drug-related side effects, hormonal changes, and immune responses commonly induce these symptoms. Chemotherapy and immunosuppressants disrupt hair growth cycles, leading to hair loss. In standard HER2-positive breast cancer therapy, mAbs are often used together with alopecia-inducing cytotoxic agents like anthracyclines and taxanes [31,32]. A report with a literature review [33] described a single-patient case with permanent alopecia following treatment with docetaxel, carboplatin, and trastuzumab. The authors noted a lack of evidence for alopecia with trastuzumab, and because carboplatin was given only as a single standard-dose infusion, docetaxel was considered the most likely causative agent.

Consistent with these observations, pharmacovigilance analyses using VigiBase showed that targeted anticancer agents are more frequently associated with dermatologic AEs such as hair color changes, whereas alopecia is predominantly reported with conventional cytotoxic agents, although sporadic signals have also been reported in pharmacovigilance databases for targeted therapies [34]. This finding suggests that the hair color changes observed in this cluster may be partly attributable to targeted therapies themselves, whereas confounding effects from concomitant cytotoxic chemotherapy might more strongly influence alopecia.

Hair that regrows after chemotherapy may differ in color, texture, thickness, or wave pattern from the patient’s original hair [35]. Pigment changes are thought to result from oxidative damage, including impaired melanin transport from follicular keratinocytes and melanocytes and melanocyte stem cell apoptosis [36]. Alterations in hair structure may also reflect changes in hair shaft keratin and asymmetric follicular growth [37,38]. Whereas a systematic review reported that approximately 15% of molecularly targeted agents are associated with alopecia [39], careful interpretation is warranted to determine whether these mAbs directly cause hair-related AEs. The involvement of concomitant cytotoxic or targeted therapies on these AEs cannot be excluded.

Although the precise causes of hair-related AEs could not be established in this study, cluster analysis enabled the visualization of AE patterns that may not be fully explained by theoretical mechanisms, providing real-world insights into the safety profiles of HER2 inhibitors.

### 3.3. Classification of AEs Based on Clustering Analysis

Based on a selection threshold of ≥1500 reports, 61 AEs were included in the clustering analysis and subsequently classified into 8 distinct clusters (Figure 2).

In this analysis, we restricted our evaluation to AEs with at least 1500 reports to minimize potential inflation of ROR values caused by low-report counts. Prior studies have shown that ROR is highly sensitive to rare events and small sample sizes, where even a few additional reports can disproportionately inflate values and lead to false-positive signals [40,41]. Therefore, we assumed that applying a high reporting threshold would mitigate such biases and improve the robustness of our signal detection.

Notably, the clusters obtained in this study were not based on conventional clinical classifications but were generated through a data-driven hierarchical clustering approach. In particular, AEs were grouped according to similarities in their lnROR profiles across HER2-targeted agents rather than based on clinical or pathophysiological similarities. This statistical grouping reflected shared reporting patterns within the FAERS database, which may reveal latent structures in AE profiles that are not readily captured by traditional clinical categorization.

- Cluster 1: abdominal pain, alopecia, cardiac failure, chills, cough, death, dyspnea, general physical health deterioration, malaise, pneumonia, pyrexia, and urinary tract infection.

This cluster comprises AEs related to systemic health deterioration (e.g., general physical health deterioration, malaise, and death), infectious symptoms (e.g., pneumonia, urinary tract infections, cough, and pyrexia), and administration-related side effects (e.g., chills and cardiac failure). As visually suggested by the heatmap in Figure 2, chills and alopecia were relatively strongly associated with mAbs.

Chills are a hallmark of infusion-related reactions, particularly during the initial administration. A strong association signal was observed with margetuximab, a chimeric anti-HER2 monoclonal antibody featuring an engineered Fc domain optimized to enhance immune activation [42,43]. In a Phase III trial combining margetuximab with chemotherapy, the overall incidence of infusion-related reactions (any grade) was 13.3%, ranking it among the top 15 most frequent AEs [44]. Within cluster 6, which includes infusion-related reactions, margetuximab showed a notable signal, suggesting that the chills reported with margetuximab may primarily be attributed to infusion-related reactions.

Alopecia also showed a notable association with mAbs, as observed in Figure 2; two hypotheses could explain this observation. First, mAbs targeting HER2 may directly affect hair follicles. However, clinical trials and basic research have not yet elucidated the causal mechanism [45]. Second, synergistic effects and confounding influences from concomitant medications must be considered. As previously discussed, molecular targeted agents are frequently administered in combination with other cytotoxic chemotherapeutic drugs. Therefore, the alopecia signal is unlikely to reflect the effects of mAbs alone and is more likely influenced by co-administered agents. In summary, whereas these findings suggest the potential involvement of mAbs in alopecia, they cannot be interpreted as evidence for a direct causal relationship. Although HER2 receptor inhibition may impact the hair follicles pharmacologically, the widespread use of combination therapies necessitates careful consideration of drug–drug interactions and confounding factors. To clarify these relationships, further investigation is needed.

**Figure 5:**
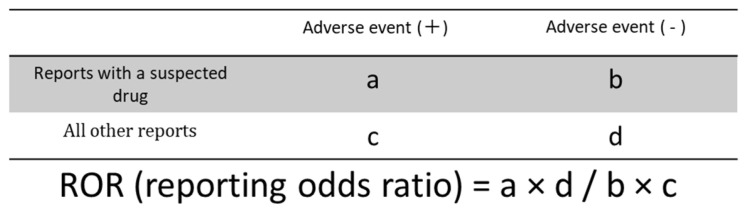
Cross-tabulation and formula used to calculate ROR for disproportionality analysis.

A recent FAERS-based analysis reported that 16.77% of AE reports for HER2 inhibitors involved infectious complications [46], highlighting the quantitative burden of infections in this drug class. Importantly, this figure represents the proportion of reports in the FAERS database and not an epidemiological incidence rate. In contrast, this study employed disproportionality analysis using lnROR (Figure 5), which does not directly evaluate reporting rates but rather indicates the relative extent to which specific AEs are reported compared to the entire database [40]. Accordingly, while previous studies quantitatively assessed the overall burden of infection-related events associated with HER2 inhibitors, our analysis identified infectious AEs as part of cluster 1, along with systemic symptoms and administration-related reactions. This reflects statistical proximity in reporting patterns rather than absolute incidence. This methodological difference may partly explain why infectious events were not emphasized in isolation. In particular, while the former approach, based on reporting rates, highlights the infection burden, the latter signal detection strategy elucidates how infectious events are positioned within the broader safety profile of HER2 inhibitors.

- Cluster 2: anxiety, arthralgia, back pain, dizziness, drug ineffective, erythema, headache, hypoesthesia, myalgia, pain, pain in extremity, paresthesia, pruritus, and rash.

This cluster included a mixture of neurological, musculoskeletal, and dermatological AEs. Notably, these events were grouped together based on statistical similarities in lnROR profiles rather than clinical or pathophysiological similarity. Neurological symptoms such as dizziness, headache, and paresthesia may reflect the limited expression of HER2 in the nervous system and the restrictive nature of the blood–brain barrier (BBB). This may reflect the limited expression of HER2 in the nervous system and the restrictive nature of the BBB.

Due to their high molecular weight, mAbs poorly penetrate the BBB, resulting in limited CNS activity. Conversely, TKIs are low-molecular weight compounds that are thought to cross the BBB more readily than high-molecular weight agents such as trastuzumab [47,48,49,50]. However, TKIs also showed low neurological impact, possibly due to their selectivity for HER2-overexpressing tumor cells, thereby preserving normal neuronal function.

ADCs share pharmacokinetic properties with trastuzumab, like slow clearance and limited distribution volume, primarily remaining confined to plasma [51]. Nonetheless, emerging evidence suggests that some ADCs have intracranial effects. Their CNS delivery mechanisms are under active investigation [52,53,54].

Musculoskeletal events such as arthralgia and myalgia may be partly explained by immune activation and cytokine release induced by HER2-targeted therapies [55], whereas dermatological symptoms like erythema and pruritus could be related to EGFR/HER family cross-inhibition [56], a mechanism known to underlie skin toxicity in targeted therapies.

Overall, these findings suggest that HER2 inhibitors may have a generally favorable CNS safety profile [57], whereas other events in this cluster (musculoskeletal and dermatological) may be attributable to distinct pharmacological or immunological mechanisms.

- Cluster 3: anemia, febrile neutropenia, neutropenia, decreased platelet count, pleural effusion, thrombocytopenia, decreased white blood cell count.

This cluster includes AEs commonly associated with myelosuppression and immunosuppression, typically resulting from cytotoxic chemotherapy. In the context of HER2 inhibitors, such effects are particularly relevant to ADCs, whose cytotoxic payloads (e.g., microtubule inhibitors or topoisomerase I inhibitors) directly impair hematopoietic stem and progenitor cells [58,59]. Monoclonal antibodies themselves may exert indirect effects through immune modulation. In contrast, TKIs aside from pyrotinib (i.e., lapatinib, tucatinib, and neratinib) showed weak associations with hematological toxicity, supporting the idea that TKIs generally carry a low risk of causing bone marrow suppression. Post-marketing evidence from EudraVigilance further indicated disproportionate reporting of hematologic toxicities with trastuzumab-based ADCs compared with their parent antibodies, aligning with the cytopenia-enriched profile observed in this cluster [60]. Pleural effusion observed in this cluster may reflect the secondary complications of immunosuppression, such as infection or disease progression, rather than a direct pharmacological effect. This pattern was consistent with the pharmacology of small-molecule TKIs, which lack cytotoxic payloads and typically show lower direct myelosuppression compared with ADCs [61]. Notably, margetuximab showed a more pronounced association strength in the heatmap in Figure 2 than other monoclonal antibodies (i.e., trastuzumab and pertuzumab). This may be attributable to its indication for HER2-positive advanced breast cancer in patients with multiple prior lines of therapy [44], placing its use in a different treatment phase from other anti-HER2 monoclonal antibodies. Such differences in treatment context may influence the degree of treatment resistance and the cumulative risk of AEs in the target patient population, which in turn could have contributed to the observed association strength in the heatmap. In addition, in our heatmap, T-DM1 showed a stronger association signal than T-DXd with hematologic toxicities, which was consistent with the report indicating that in EudraVigilance, T-DM1 had a slightly higher disproportionality measure for blood and lymphatic system disorders than T-DXd [60].

- Cluster 4: asthenia, constipation, decreased appetite, dehydration, diarrhea, epistaxis, fatigue, malignant neoplasm progression, nausea, stomatitis, vomiting, and decreased weight.

This cluster encompasses gastrointestinal dysfunction, nutritional decline, and general systemic deterioration. Unlike cluster 3, TKIs such as lapatinib, tucatinib, and neratinib showed stronger correlations with these events.

Diarrhea was associated with all TKIs, including pyrotinib, which irreversibly inhibits HER family members. Phase II/III trials have reported a high incidence of diarrhea (83% for pyrotinib [62]; 97% for neratinib [14]). This toxicity is likely mediated by EGFR inhibition in the gastrointestinal tract, which disrupts mucosal integrity [24]. Experimental and clinical data have indicated that EGFR signaling supports epithelial restitution and tight-junction integrity in the gut; inhibition can impair barrier function and favor secretory diarrhea [63,64]. Other events in this cluster, such as stomatitis, nausea, and vomiting, may also result from epithelial barrier injury and inflammation, whereas anorexia, fatigue, and weight loss can be driven by malabsorption and systemic cytokine responses. These features also overlap with cancer cachexia, a multifactorial syndrome characterized by the ongoing loss of skeletal muscle mass and systemic inflammation [65,66]. This was consistent with canonical models of therapy-induced mucosal injury and oral mucositis, which involve epithelial damage, inflammatory amplification, and impaired healing [67]. Malignant neoplasm progression included in this cluster suggests that some signals reflect the underlying disease course rather than direct drug toxicity. Severe diarrhea may lead to fluid and electrolyte loss, contributing to dehydration and nutritional decline. Dose escalation and prophylactic antidiarrheal measures can reduce its severity [68]. Beyond clinical trials, VigiBase analyses of EGFR-targeted therapies have repeatedly highlighted digestive AEs—particularly diarrhea—as prominent signals in real-world reports, reinforcing the biological plausibility for TKI-associated gastrointestinal toxicity [69]. For neratinib, proactive management strategies tested in the CONTROL trial—such as initial dose escalation with loperamide as needed—significantly improved tolerability and reduced high-grade diarrhea [70].

Overall, these gastrointestinal and systemic toxicities underscore the importance of proactive supportive measures, including prophylactic antidiarrheal therapy, nutritional support, and close monitoring, to ensure treatment adherence and optimal patient QoL.

- Cluster 5: hair color changes, hair disorders, abnormal hair texture, and madarosis.

This cluster exclusively comprises hair-related AEs, with strong associations to mAbs, as discussed in Section 3.2. These events may reflect post-chemotherapy hair regeneration, immunological effects, or indirect pharmacologic consequences. These events may not solely reflect the pharmacologic effects of mAbs but could also be influenced by concomitant cytotoxic therapies known to impair follicular cycling.

- Cluster 6: infusion-related reactions and ILD.

These events were more prevalent with intravenously administered agents, such as trastuzumab, pertuzumab, margetuximab, T-DXd, and T-DM1.

Infusion-related reactions are typically mediated by the release of cytokines (e.g., IL-6 and TNF-α) and complement activation during initial antibody exposure, which explains their predominance among intravenously administered agents [71].

ILD was particularly associated with T-DXd and is a known AE with a reported high frequency [11,72]. Our analysis confirmed the strong positive signal with T-DXd, whereas neratinib exhibited a strong negative correlation. These trends are consistent with prior studies evaluating ILD in cancer therapies [73]. Mechanistically, ILD with T-DXd may result from off-target toxicity of the topoisomerase I payload or immune-mediated lung injury involving macrophage activation, as suggested by preclinical and clinical investigations [74,75,76].

- Cluster 7: myelosuppression.

This cluster consisted solely of myelosuppression, a condition commonly observed in chemotherapy, particularly when cytotoxic agents impair hematopoietic stem cells in the bone marrow, thereby suppressing blood cell production. In our analysis, myelosuppression was associated with ADCs and mAbs. Among TKIs, pyrotinib showed a strong positive association, whereas other TKIs exhibited negative correlations.

TKIs carry a relatively low risk of bone marrow suppression; however, certain TKIs—such as imatinib [77], nilotinib [78], and dasatinib [79], used in chronic myeloid leukemia treatment—present hematologic toxicity. Pyrotinib irreversibly inhibits multiple HER family members (EGFR, HER2, and HER4) [27], a pharmacological property that may contribute to its hematologic profile, although direct comparative evidence with conventional TKIs is limited. The observed myelosuppression with pyrotinib and ADC-containing regimens [80] could also be influenced by concomitant chemotherapy, highlighting the importance of carefully distinguishing between drug-specific and combination-related effects.

These findings may suggest that pyrotinib possesses a unique pharmacological mechanism and safety profile distinct from that of conventional TKIs.

As noted previously, pyrotinib is often an alternative to ADCs, and this must be taken into consideration. Therefore, further investigation is necessary to elucidate the underlying causes of myelosuppression and comprehensively assess pyrotinib’s safety profile.

- Cluster 8: breast cancer metastatic, cardiotoxicity, disease progression, decreased ejection fraction, hypokalemia, metastases to the central nervous system, mucosal inflammation, peripheral neuropathy, and palmar–plantar erythrodysesthesia syndrome

In contrast to cluster 2, cluster 8 exhibited strong associations with AEs across the examined agents. This cluster likely represents AEs commonly reported in association with HER2-targeted therapies.

In HER2-positive breast cancer, central nervous system metastases occur in up to 50% of patients [81,82], depending on prior therapy and disease stage, and this phenomenon is frequently encountered in clinical practice. In our analysis, central nervous system metastases showed strong associations with all examined HER2-targeted agents. Drugs with HER2-inhibitory activity, including mAbs, ADCs, and TKIs, have been associated with cardiotoxicity, possibly related to HER2 expression in cardiomyocytes [6,15,16,83,84]. HER2 signaling plays a critical role in cardiomyocyte survival through pathways such as PI3K–Akt and MAPK; inhibition of these protective pathways may impair cellular resilience under stress and contribute to cardiac dysfunction [85]. Analysis of EudraVigilance has identified disproportionate reporting signals for trastuzumab-based ADCs, including trastuzumab, emtansine, and trastuzumab deruxtecan, reinforcing the robustness of cardiotoxicity signals across pharmacovigilance platforms [60]. This aligns with clinical evidence summarized by Curigliano et al. (2016) [86], which established HER2-targeted therapy–related cardiotoxicity as a well-recognized safety profile.

Moreover, these drugs are frequently administered in conjunction with other cardiotoxic therapies, like anthracyclines [87], which may further exacerbate cardiac risks.

In this analysis, decreased ejection fraction was associated with all HER2 inhibitors except pyrotinib, whereas cardiotoxicity showed only a weak correlation with neratinib. Notably, many clinical trials exclude patients with pre-existing cardiac abnormalities, suggesting that the incidence of cardiotoxicity may be underestimated relative to real-world clinical practice. However, this study utilized the FAERS database to analyze a broader and more diverse patient population, allowing AE patterns to be reflected under real-world medical conditions. Given the risk of cardiac issues during HER2-targeted therapy, the FDA has recommended baseline cardiac screening and at intervals throughout treatment [88].

Palmar–plantar erythrodysesthesia syndrome, also known as hand–foot syndrome (HFS), was associated with all agents except T-DXd. HFS is commonly induced by fluoropyrimidine agents such as capecitabine [89]. In a clinical trial in patients with residual HER2-negative invasive breast cancer evaluating adjuvant capecitabine following preoperative chemotherapy, the incidence of HFS in the capecitabine arm was as high as 73.4% [90]. Many HER2 inhibitors associated with HFS are commonly administered with capecitabine [12,13,14,29,44,48,49,50,91]. The pathophysiology of HFS is thought to involve direct toxic effects of fluoropyrimidine metabolites on capillary endothelial cells, dermal microvasculature, and eccrine glands in the palms and soles, leading to inflammation, vascular damage, and keratinocyte injury [92].

These findings highlight the need to carefully consider the influence of combination therapies involving capecitabine.

### 3.4. PCA

In this study, hierarchical clustering analysis and PCA were jointly applied to characterize the relationships between HER2-targeted drugs and AEs. Cluster analysis enabled the classification of drugs with similar AE profiles, facilitating pattern recognition across the entire dataset. However, clustering alone may overlook the underlying structure and directional variance within the data [93]. To address this, PCA was used as a dimensionality reduction technique to summarize multivariate data into principal components, thereby enhancing interpretability while minimizing information loss [94].

Principal component 1 was positively correlated with lnROR (Figure 4). Moreover, as shown in the PCA score plot (Figure 3b), cluster 8, which demonstrated strong associations with AEs across all evaluated HER2-targeted drugs, was distributed in the positive direction along the principal component 1 axis. In contrast, cluster 2, characterized by generally weaker associations, was plotted in the negative direction. Both clusters were discussed in detail in Section 3.3. This spatial separation supports the interpretation that principal component 1 reflects the overall risk of developing AEs likely to be induced by HER2 inhibitors.

Principal component 2 showed positive correlations with TKIs like lapatinib (r = 0.450), neratinib (r = 0.464), and tucatinib (r = 0.667), while demonstrating negative correlations with mAbs including pertuzumab (r = −0.542), trastuzumab (r = −0.567), and margetuximab (r = −0.367). Thus, principal component 2 may reflect differences in the mechanisms of action of these drugs. Specifically, intracellular HER2 tyrosine kinase inhibition is plotted toward the positive axis, whereas extracellular HER2-targeted antibody effects are plotted toward the negative axis.

The observation that AE reporting patterns are expressed in principal component 2, based on differences in mechanisms of action, suggests that the high selectivity of molecularly targeted agents is strongly reflected in their AE profiles.

ADCs, including T-DXd and T-DM1, have hybrid mechanisms combining HER2 targeting via the antibody portion with cytotoxic payload delivery through linkers [95,96]. Due to these unique characteristics, ADCs may occupy a neutral position, appearing neither fully aligned with low-molecular-weight TKIs (positive axis), which directly inhibit intracellular signaling, nor with mAbs (negative axis), which act through immune-mediated receptor targeting. This suggests that their hybrid mechanism could plausibly account for the intermediate positioning observed in our analysis.

Although pyrotinib is classified as a TKI, it has multiple associations with AEs typically observed with cytotoxic chemotherapeutic agents, such as bone marrow suppression (Figure 2 and Figure 3). This may differ from that of other TKIs, resulting in a neutral position in the PCA.

Principal component 3 was positively correlated with neratinib (r = 0.521) and negatively correlated with T-DXd (r = −0.440) and T-DM1 (r = −0.325). In the plot, the positive axis was associated with AEs involving localized tissues such as skin and mucosa. Conversely, the negative axis captured AEs commonly reported for ADCs, including decreased platelet count and ILD (Figure 4). This suggests that principal component 3 may distinguish AE profiles between pure molecular targeted agents and ADCs.

Off-target toxicity, which refers to toxic effects unrelated to the antibody’s target molecule and often constitutes dose-limiting toxicities [97], is a key issue in ADC safety assessments. The cytotoxic payloads used in ADCs are generally highly potent and often too toxic to be used as standalone drugs [98]. ADCs are designed with linkers that stabilize the payload in serum and promote rapid clearance outside the tumor [99,100]. The target-dependent mechanisms of ADCs enhance tissue selectivity and cytotoxic efficacy toward cancer cells during drug action, while simultaneously reducing, but not eliminating, the systemic toxicity associated with the cytotoxic payload.

Off-target toxicity may result from premature linker cleavage [101] or unintended ADC uptake into nontarget cells [102]. Thrombocytopenia observed with T-DM1 may occur via Fc receptor-mediated uptake into megakaryocytes, followed by payload-induced cellular injury, as demonstrated in in vitro studies [102].

T-DXd is composed of trastuzumab conjugated via a cleavable tetrapeptide linker to a topoisomerase I inhibitor payload [99,103]. As previously described, the most serious AE associated with T-DXd is ILD through an undetermined mechanism. ILD with T-DXd may result from off-target toxicity following payload release or immune-mediated lung injury involving macrophage activation [104]. Preclinical studies in monkeys revealed that T-DXd is preferentially localized in macrophages rather than pulmonary epithelial cells [76].

Our observations suggest that AEs associated with ADCs may be related to off-target effects. Cytotoxic AEs induced by the payload are observed as negative loadings in principal component 3, implying that this axis represents toxicity specific to molecularly targeted agents.

Based on the structure of principal components 1 through 3, we can summarize the tendencies of drugs and their corresponding AEs. TKIs, such as neratinib and lapatinib, showed strong associations with diarrhea, whereas mAbs, like trastuzumab and pertuzumab, were significantly associated with hair-related AEs. To the best of our knowledge, this is the first report in the literature to extract AE profiles using cluster analysis and PCA applied to SRS data.

While ADCs such as T-DM1 and T-DXd have structurally incorporated monoclonal antibodies, their pharmacological activity is primarily driven by their payload. The payload may induce AEs like decreased platelet count and ILD, which are distinct from AEs specific to non-ADC molecular targeted agents (e.g., trastuzumab and neratinib). Thus, PCA successfully demonstrated differences in AEs based on drug characteristics (Figure 3c,d). Through this comprehensive evaluation, each principal component captured clinically relevant, drug-specific AE profiles, providing valuable insights to predict side effects and formulate management strategies during treatment selection.

### 3.5. Limitations and Strengths

This study has several limitations. First, because the FAERS database is based on spontaneous reports, it may be subject to reporting bias and contain incomplete or inaccurate information. The number of reports can vary depending on the reporter’s interest level, and not all AEs are consistently documented. Mild AEs may be underreported, whereas severe events are more likely to be frequently captured. Furthermore, the total number of patients receiving HER2 inhibitors is unknown, which precludes an accurate estimation of the true incidence of AEs. Therefore, our results indicate associations between drugs and AEs but do not directly demonstrate causality. To establish causal relationships, more rigorous approaches, including randomized controlled trials or large-scale observational studies, are required.

Moreover, confounding factors, including underlying diseases, concomitant medications, and treatment duration, were not included in this analysis but may impact the occurrence of AEs. Thus, any interpretation of the results must account for both qualitative and quantitative limitations inherent to SRSs.

Moreover, given that the ROR calculation process is susceptible to substantial bias, direct comparisons between numerical values are constrained. Accordingly, our study was conducted based on the hypothesis that limiting the analysis to the top 10% of AEs by report frequency may ensure reliability in ROR-based comparisons.

Despite these limitations, our analysis successfully captured the real-world patterns of AE manifestation during clinical use—for example, the association between hair-related AEs and monoclonal antibodies identified through cluster analysis. Additionally, PCA enabled us to estimate latent components that may influence the AEs associated with HER2 inhibitors.

## 4. Materials and Methods

### 4.1. Data Table Construction

To perform a comprehensive analysis of AEs associated with HER2 inhibitors, we utilized the FAERS database, which covers cases reported from January 2004 to September 2024 (as of the October 2024 release) [105]. FAERS comprises seven data tables: DRUG, REAC, DEMO, Outcome (OUTC), Report Sources (RPSR), Indication (INDI), and Therapy (THER). Data curation, including coding, mapping, and cleaning, was conducted by ArkMS Inc. (https://www.arkms.co.jp/, accessed on 13 June 2025). For this analysis, we constructed an integrated analysis table by joining the DRUG, REAC and DEMO tables using the primary ID. The DRUG table classified the reported drugs into four categories: primary suspected drug, secondary suspect drug, concomitant drugs, and interactions.

To ensure the reliability of the analysis, duplicate reports were removed before data integration. In particular, from the DRUG and REAC tables, we extracted only the primary ID together with the corresponding drug name or AE term. We removed duplicate entries based on identical primary ID–term combinations. In addition, from the DEMO table, we extracted the primary ID along with demographic and reporting information, including the FDA report date and age, age unit, gender, body weight, weight unit, and reporting country. The DRUG, REAC, and DEMO tables were merged using a full outer join on the primary ID, and records with missing values in either the DRUG or REAC table were excluded. Age values reported in days, weeks, months, or decades were converted to years. For decade-coded ages, the midpoint of each decade was assigned (years = 10 × value + 5). Body weight values reported in pounds were converted to kilograms. These standardized values were used for descriptive statistics and tabulations.

This study was based on anonymized data obtained from a publicly accessible database; therefore, the Ethics Committee of Meiji Pharmaceutical University waived the requirement for ethical approval and informed consent.

### 4.2. Terminology for AEs and Targeted Drugs

The Medical Dictionary for Regulatory Activities (MedDRA) is an international medical terminology developed by the International Conference on Harmonization of Drug Regulations (ICH) to promote the global sharing of regulatory information on medical products for human use. MedDRA follows a five-level hierarchical structure consisting of System Organ Class (SOC), High Level Group Terms (HLGT), High Level Terms (HLT), Preferred Terms (PT), and Lowest Level Terms (LLT). In this study, AEs were coded using the PT of MedDRA version 27.1.

### 4.3. Selection and Exclusion Criteria

Selection Criteria

Drugs and AEs were included in the analysis if they met the following conditions:

- Drugs with HER2-inhibitory activity approved for breast cancer treatment.
- AEs reported in association with the above drugs in the FAERS database.
- AEs with the number of reports exceeding approximately 10% of the maximum count observed (i.e., >1500 reports).
- PTs coded using MedDRA version 27.1.
- Drugs designated as primary or suspect in the FAERS DRUG table.
- Primary IDs corresponding to HER2 inhibitors were fully retained after merging.

Exclusion Criteria

The following conditions were applied to refine the dataset:

- Drugs not classified as HER2-targeted agents or not approved for breast cancer treatment.
- AEs with 1500 or fewer total reports.
- AE terms deemed nonclinical or nonspecific, such as “no adverse event” or “off-label use.”
- Concomitant and interaction drugs listed in the FAERS DRUG table.
- Records with missing values in the DRUG or REAC tables after merging.

### 4.4. Outcomes

The primary outcomes of this study were to (1) identify the major AEs associated with HER2 inhibitors based on disproportionality analysis using RORs and (2) comprehensively characterize the patterns of these AEs by applying data mining approaches, including cluster analysis and PCA, to elucidate the statistical structures and class-specific tendencies of AE profiles.

The secondary outcomes included reassessing known safety signals and detecting potential novel, previously unrecognized safety signals.

### 4.5. Disproportionality Analysis of AEs Associated with HER2 Inhibitors

We used the ROR, its 95% confidence interval, and the *p*-value derived from Fisher’s exact test to assess the relationship between HER2 inhibitors and AEs. When the contingency tables contained zero cells, the ROR calculation was not feasible, and the estimation became unstable. To correct for potential bias, we applied the Haldane–Anscombe correction by adding 0.5 to each cell value [106].

We constructed 2 × 2 contingency tables to investigate the associations between HER2 inhibitors and all reported AEs (Figure 5). Based on these tables, RORs were calculated to estimate the strength of associations. Subsequently, the ROR values and Fisher’s exact test were used to evaluate the statistical significance of AEs associated with HER2 inhibitors.

### 4.6. Construction of the Data Matrix for Clustering and PCA

To investigate the associations between HER2 inhibitors and AEs, we used the lnROR as a quantitative indicator. A data matrix of lnROR values was constructed for AEs extracted according to the criteria described in Section 4.3. Nine agents were included: TKIs (lapatinib, neratinib, tucatinib, pyrotinib); mAbs (trastuzumab, pertuzumab, margetuximab); and ADCs (T-DM1, T-DXd). The resulting lnROR matrix was incorporated into Table S2. lnROR was used to enhance the interpretability and statistical properties of the data. In particular, log transformation reduces skewness, stabilizes variance, and symmetrizes the distribution of ROR values, thereby facilitating downstream analyses such as clustering and PCA.

### 4.7. Hierarchical Clustering Analysis

To classify the AEs associated with HER2 inhibitors, hierarchical clustering analysis (Ward’s method) was performed based on the lnROR matrix constructed in Section 4.6.

To ensure objective cluster determination, we selected the number of clusters by examining the distance graph (dendrogram height plot) and identifying the inflection point where the slope sharply increased. This approach adhered to a data-driven criterion rather than an arbitrary choice of cluster count.

Ward’s method, a hierarchical clustering algorithm, was employed due to its ability to minimize the within-cluster variance during each agglomeration step [107]. By reducing intracluster variance, this method facilitates the formation of internally homogeneous clusters. While Ward’s method is not specifically designed to handle outliers, and extreme values can still influence results. However, compared with other linkage methods, the Ward’s method may be less affected by extreme values [108].

Furthermore, because cluster merging is based on variance, the resulting hierarchical structure is clearly defined and easily interpretable through dendrogram visualization.

### 4.8. PCA

A principal component is a newly derived variable created through PCA, which transforms a set of correlated variables into a smaller number of uncorrelated components. Each principal component represents a linear combination of the original variables and captures a specific pattern of variation within the dataset. The first principal component explains the largest amount of variance, followed by the second, third, and so on. In this study, principal components were used to summarize the relationships between HER2 inhibitors and AE profiles.

PCA was conducted using the lnROR matrix described in Section 4.6, with the analysis based on a correlation coefficient matrix.

Correlation coefficients (r) derived from the PCA loading matrix were used as quantitative indicators to assess the relationship between each drug and its respective principal components, reflecting the extent to which each drug contributes to the variation captured by each component.

To evaluate the multivariate relationships between HER2 inhibitors and AEs, the HER2-targeted drugs were treated as a unified group. The group-wise lnROR values were utilized to assess correlations with principal components. Specifically, lnROR values across 61 AEs were plotted against the principal components, and regression lines were fitted to visually examine the relationship between AE trends and each component (Figure 4).

### 4.9. Statistical Analysis

The database processing, descriptive statistics, cluster analysis, and PCA were performed using JMP Pro 18.2 (SAS Institute Inc., Cary, NC, USA). To construct the 2 × 2 contingency tables, calculate RORs (Figure 1), derive 95% confidence intervals, and conduct Fisher’s exact tests, we utilized the Python 3.12.3 environment with the pandas (v2.3.1) and SciPy packages (v1.13.1). All statistical significance thresholds were set at *p* < 0.05.

## 5. Conclusions

In this study, we focused on HER2 inhibitors approved for breast cancer treatment and conducted disproportionality analysis to identify AEs with high RORs. Furthermore, using data mining techniques such as cluster analysis and PCA, we comprehensively characterized the statistical structure and trends of AE profiles associated with HER2 inhibitors.

Cluster analysis based on lnROR enabled classification of AEs which revealed that hair-related events showed a particularly strong association with mAbs. PCA demonstrated that principal component 2 effectively distinguished mAbs from TKIs, whereas principal component 3 suggested a potential separation between pure molecular targeted agents and ADCs.

Based on these findings, the trends in AE occurrence associated with HER2 inhibitors may be influenced by their mechanisms of action, as suggested by statistical analyses using data mining techniques such as cluster analysis and PCA. Furthermore, the frequently reported AEs observed with ADCs may be attributable to their payload components.

In addition to reassessing known safety signals—such as cardiotoxicity, diarrhea associated with TKIs, and ILD linked to T-DXd—we captured previously underrecognized AEs, including skin disorders primarily related to hair.

These findings enable a visual understanding of the AE trends linked to each HER2 inhibitor used during the treatment of HER2-positive breast cancer. The comprehensive visualization of drug-specific AE features in real-world settings may facilitate intuitive grasp and practical handling of these agents by medical professionals. Additionally, examining real-world safety profiles for individual drugs may promote intuitive comprehension among medical professionals when handling these agents in clinical practice. Additionally, similar AE patterns across different agents may encourage shared strategies in side effect management, contributing to improved strategic planning. Ultimately, these insights are expected to facilitate treatment approach optimization focusing on QoL enhancement and improve both drug selection and AE management.

## Figures and Tables

**Table 1:** Baseline Characteristics of Patients Reporting Adverse Events Associated with HER2 Inhibitors.

Characteristics		No.	(%)
Number of Reports		96,222	
Sex			
	Data available	82,416	
	Female	76,736	93.11%
	Male	5108	6.20%
	Unknown	572	0.69%
Age (years)			
	Data available	57,608	
	<30	675	1.17%
	30–39	4618	8.02%
	40–49	11,135	19.33%
	50–59	17,031	29.56%
	60–69	14,645	25.42%
	70–79	7626	13.24%
	80 or older	1878	3.26%
	Median (IQR)	57 (48–66)	
Body weight (kg)			
	Data available	27,265	
	<40	379	1.39%
	40–49	2382	8.74%
	50–59	6365	23.35%
	60–69	7140	26.19%
	70–79	4705	17.26%
	80–89	3067	11.25%
	90 or more	3227	11.84%
	Median (IQR)	65 (56–78)	
Reporting countries (Top 5)			
	Data available	95,625	
	United States	37,984	39.72%
	China	6111	6.39%
	Japan	5945	6.22%
	United Kingdom	5646	5.90%
	Canada	5516	5.77%

**Table 2:** Number of adverse event (AE) reports for nine HER2 inhibitors across 61 selected AE categories.

Drug	Number of Reports	(%)	First Report Date
Monoclonal antibodies (mAbs)	141,878	(69.14)	
Trastuzumab	101,528	(49.48)	7 January 2004
Pertuzumab	40,264	(19.62)	29 October 2004
Margetuximab	86	(0.04)	9 March 2021
Antibody–drug conjugates (ADCs)	20,711	(10.09)	
Trastuzumab emtansine (T-DM1)	13,613	(6.63)	23 September 2011
Trastuzumab deruxtecan (T-DXd)	7098	(3.46)	22 January 2020
Tyrosine kinase inhibitors (TKIs)	42,607	(20.76)	
Lapatinib	27,103	(13.21)	24 August 2004
Tucatinib	9259	(4.51)	4 November 2004
Neratinib	5535	(2.70)	31 July 2009
Pyrotinib	710	(0.35)	20 December 2018

## Data Availability

Data are contained within the article and Supplementary Materials.

## Supplementary Materials

The following supporting information can be downloaded at: https://www.mdpi.com/article/10.3390/ph18101510/s1, Table S1: Statistical summary of the top 61 AEs associated with HER2 inhibitors based on reporting odds ratios (RORs); Table S2: lnROR matrix for 61 AEs and HER2 inhibitor clusters, including mAbs (trastuzumab, pertuzumab, and margetuximab), ADCs (T-DM1 and T-DXd), and TKIs (lapatinib, neratinib, tucatinib, and pyrotinib).

## Abbreviations

The following abbreviations are used in this manuscript:

AE	Adverse event
FDA	Food and Drug Administration
ILD	Interstitial lung disease
JSPS	Japan Society for the Promotion of Science
lnROR	Natural logarithm of the reporting odds ratio
PCA	Principal component analysis
QoL	Quality of life
RORs	Reporting odds ratios
SRSs	Spontaneous reporting systems
mAbs	Monoclonal antibodies
ADCs	Antibody–drug conjugates
TKIs	Tyrosine kinase inhibitors
